# Surveillance for Antibiotic-Resistant *E. coli* in the Salish Sea Ecosystem

**DOI:** 10.3390/antibiotics10101201

**Published:** 2021-10-02

**Authors:** Alexandria Vingino, Marilyn C. Roberts, Michelle Wainstein, James West, Stephanie A. Norman, Dyanna Lambourn, Jeffery Lahti, Ryan Ruiz, Marisa D’Angeli, Scott J. Weissman, Peter Rabinowitz

**Affiliations:** 1 Department of Environmental and Occupational Health Sciences (DEOHS), University of Washington, Seattle, WA 98105, USA or alexandriavingino@gmail.com (A.V.); peterr7@uw.edu (P.R.); 2 Woodland Park Zoo, Seattle, WA 98105, USA; michelle@creoi.org; 3 Washington Department of Fish and Wildlife, P.O. Box 43200, Olympia, WA 98504, USA; james.west@dfw.wa.gov (J.W.); dyanna.lambourn@dfw.wa.gov (D.L.); 4 Marine-Med: Marine Research Epidemiology, Veterinary Medicine, Bothell, WA 98021, USA; stephanie@marine-med.com (S.A.N.); marisa.dangeli@doh.wa.gov (M.D.); 5 Washington State Department of Health, Shoreline, WA 98105, USA; jeff.lahti@doh.wa.gov (J.L.); ryan.ruiz.85@gmail.com (R.R.); 6 Division of Infectious Disease, Seattle Children’s Hospital, Seattle, WA 98105, USA; scott.weissman@seattlechildrens.org

**Keywords:** antibiotic resistance, *E. coli*, marine mammals, environment, river otters

## Abstract

*E. coli* was isolated from the Salish Sea (Puget Sound) ecosystem, including samples of marine and fresh water, and wildlife dependent on this environment. *E. coli* isolates were assessed for phenotypic and genotypic resistance to antibiotics. A total of 305 *E. coli* isolates was characterized from samples collected from: marine water obtained in four quadrants of the Salish Sea; select locations near beaches; fresh water from streams near marine beaches; and fecal samples from harbor porpoises (*Phocoena phocoena*), harbor seals (*Phoca vitulina*), river otters (*Lontra canadensis*), and English sole (*Parophrys vetulus*). Isolates were evaluated using antimicrobial susceptibility typing, whole-genome sequencing, *fumC*, and multilocus sequence typing. Resistance and virulence genes were identified from sequence data. Of the 305 isolates from Salish Sea samples, 20 (6.6%) of the *E. coli* were intermediate, and 31 (10.2%) were resistant to ≥1 class of antibiotics, with 26.9% of nonsusceptible (resistant and intermediate resistant) *E. coli* isolates from marine mammals and 70% from river otters. The proportion of nonsusceptible isolates from animals was significantly higher than samples taken from marine water (*p* < 0.0001). A total of 196 unique STs was identified including 37 extraintestinal pathogenic *E. coli* (ExPEC)-associated STs [ST10, ST38, ST58, ST69, ST73, ST117, ST131, and ST405]. The study suggests that animals may be potential sentinels for antibiotic-resistant and ExPEC *E. coli* in the Salish Sea ecosystem.

## 1. Introduction

The anthropogenic use of antibiotics in clinical, agricultural, and community settings has contributed to the spread of antibiotic-resistant bacteria (ARB) throughout the world, affecting many ecosystems [1]. We report on an exploratory study characterizing multiple samples from the Salish Sea ecosystem including water and animals to determine the level of antibiotic-resistant *E. coli* within various sources throughout the Salish Sea environment and its wildlife.

Antibiotic-resistant *E. coli* have been previously identified in wildlife primarily from land animals and birds [2]. By contrast, most studies on marine animals have looked at bacteria that cause diseases or are relatively easy to isolate, such as respiratory bacteria rather than normal intestinal flora such as *E. coli* [3,4]. Other studies have focused on ARB associated with fish in aquaculture settings, especially in the context of antibiotic treatment, but rarely do these studies include *E. coli* [5,6].

In previous studies, *E. coli* has been isolated over a wide global area in diverse ecosystems, organisms, and locations, making it an ideal marker organism [7]*. E. coli* has a large number of sequence types (STs) identified using multilocus sequence type methods (MLST) [7], many of which have been described in terms of pathogenesis and virulence. It also has a number of well-characterized antimicrobial-resistance genes (https://ege.cbs.dtu.dk accessed on 1 March 2021) and virulence factors [8].

## 2. Results

### 2.1. E. coli Isolates and Antibiotic Resistance

**Figure 1:**
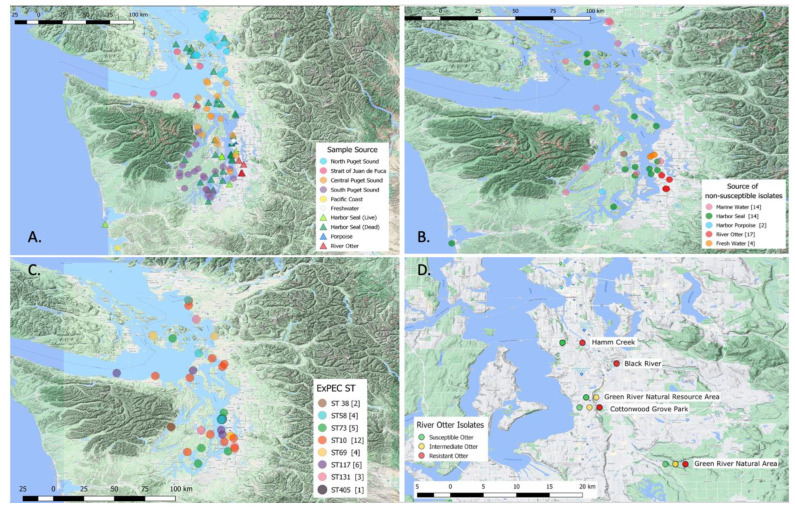
Maps of (**A**): all *E. coli* isolates by sample source; (**B**): resistant and intermediate *E. coli* isolates by sample source; (**C**): ExPEC STs of *E. coli* by location; (**D**): river otter *E. coli* isolate results.

From the total 551 samples collected, 305 isolates were selected for further study using phenotypic and genotypic antibiotic-resistance analysis (Table 1). A total of 212 *E. coli* isolates was analyzed for resistance from the four quadrants of marine water. All fresh water (*n* = 5) samples and marine water by beaches samples (*n* = 3) were included in analysis. Fifty fish were cultured with two *E. coli* positive samples. A total of 24 *E. coli* from 40 river otter samples was selected for further characterization. Thirty-five isolates from dead seals and all seven harbor porpoise isolates were included in the analysis, while 17 *E. coli* from live harbor seals were also included (Table 1, Figure 1A).

The correlation between resistant phenotypes and genes varied by antibiotic. Of the 31 *E. coli* isolates phenotypically resistant to at least one tested antibiotic, 25 (80.6%) carried between one and four genes coding for resistance to different classes of antibiotics, while six (19.4%) did not carry resistance genes (Table 2). Twenty (6.6%) *E. coli* were phenotypically intermediate resistant with two (10%) carrying known resistance genes. All phenotypic tetracycline intermediate and resistant isolates (*n* = 16) carried *tet*(A), *tet*(B), or both *tet* genes (Table 2). Fifteen *E. coli* isolates were phenotypically resistant to β-lactam antibiotics, of which 12 (80%) carried a *bla* gene. Among fourteen isolates that were sulfonamide resistant, seven (50%) carried a *sul* gene and two did not, while six (42.9%) carried both *sul* and *dfr* genes. By contrast, fourteen isolates carried aminoglycoside-resistance genes by whole-genome sequencing (WGS) analysis, but only two (28.6%) were phenotypically resistant (Table 2). Four *E. coli* were phenotypically resistant to fluoroquinolones and two phenotypically intermediate resistant; eight *E. coli* carried fluroquinolone-resistant genes, and of those, three (37.5%) had mutations by WGS analysis (Table 2). We did not test for macrolides or lincosamides, although we had two river otter *E. coli* isolates which carried the *lnu*(F) gene, and one fresh water isolate carrying *mph*(A). Similarly, most isolates were not tested for chloramphenicol or florfenicol, but one isolate from fresh water carried the chloramphenicol *catA1* gene and one from live seal scat carried the *floR* gene (Table 2).

For marine water sources, the proportion of *E. coli* isolates from the four quadrants of the Salish Sea resistant to ≥1 antibiotic ranged from 0–8.2%, while intermediate resistance ranged from 0–6.1% (Table 1). All three *E. coli* isolates from marine water near beaches were susceptible. Of the five fresh water samples, three (60%) were resistant, one (20%) showed intermediate resistance, and one (20%) was susceptible. Though the number of fresh water isolates was small, this level of *E. coli* resistance is similar to previous studies of fresh water [9,10].

Of 35 *E. coli* isolates from dead harbor seals, 3 (8.6%) were resistant, and 6 (17.1%) were intermediate to ≥1 antibiotic, and among 17 live seal isolates, 5 (29.4%) were resistant, and none were intermediate (Table 1). Two of seven isolates (28.6%) from harbor porpoises were intermediate resistant (Table 1).

The proportions of nonsusceptible *E. coli* were the highest among isolates obtained from river otter fecal samples (Table 1) with 13 (54. 2%) resistant and 4 (16.7%) intermediate resistant (Figure 1B,D). Four (30.8%) of the resistant isolates did not carry known resistance genes (Table 2). The nonsusceptible *E. coli* were mapped along the river and appeared to lack any obvious pattern across the industrial, suburban, and rural geographic zones (Figure 1D).

### 2.2. MLSTs and ExPEC Strains

We identified 196 unique STs including 139 ST represented by a single isolate, 29 ST with two isolates, 10 ST with three isolates, six ST with four isolates, five ST with five isolates, three ST with six isolates, two ST with seven isolates, and one ST represented by eight isolates. The most common ST was ST10, represented by 12 isolates. Among 37 isolates, eight ExPEC STs were identified: ST10, ST38, ST58, ST69, ST73, ST117, ST131, and ST405 (Figure 1C). These STs have been previously associated with human disease and were further examined (Table 3) [11,12]. ST10 is also widely found around the world [13]. Twenty-one (56.7%) of the ExPEC *E. coli* were isolated from marine water samples (Table 3). Others were isolated from fresh water, marine water from beaches, live harbor seals, and river otter fecal samples. Eleven (29.7%) were resistant, including 25% of ST10 (*n* = 3), 50% of ST58 (*n* = 2), 25% of ST69 (*n* = 1), 100% of ST405 (*n* = 1), 33% of ST117 (*n* = 2), and 60% of ST131 (*n* = 2) (Table 3). No ExPEC isolates were found in sole, harbor porpoises, or dead seals (Table 3).

### 2.3. Comparison of Susceptibility Rates

There were no statistically significant differences in the proportions of antibiotic-resistant *E. coli* from the four quadrants of Puget Sound (*p* = 0.089). Similarly, there were no statistically significant differences in proportions of nonsusceptibility (intermediate or resistant) and susceptibility among *E. coli* from the four quadrants of the Puget Sound (*p* = 0.148). Compared to marine water samples, wildlife sources (harbor seal, harbor porpoise, and river otter) of *E. coli* had significantly higher proportions of resistant (*p* < 0.0001; odds ratio (OR) = 8.88; 99.2% CI: 2.67–35.29) and nonsusceptible isolates (*p* < 0.0001; OR = 5.3; 95% CI: 2.21–13.40). When only marine mammal samples (river otter excluded) were compared to marine water samples, marine mammal isolates were significantly more likely to be nonsusceptible (*p* = 0.005; OR = 3.01; 99.2% CI: 1.04–8.58), as compared to marine water isolates. In comparing the proportion of antibiotic-resistant E. coli between marine mammals to that of marine water, the odds of detecting resistance in marine mammals was four times that of in marine water (*p* = 0.010; OR: 3.95, 99.2% CI: 0.83–18.84).

### 2.4. Phylogenetic Trees for ST10 and ST73

**Figure 2:**
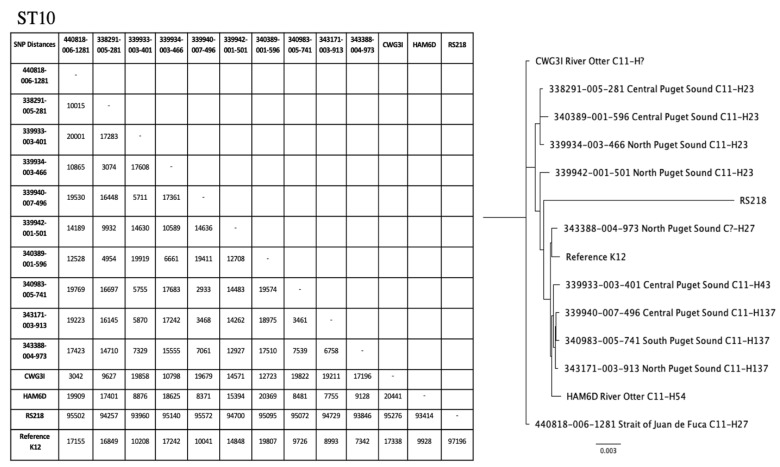

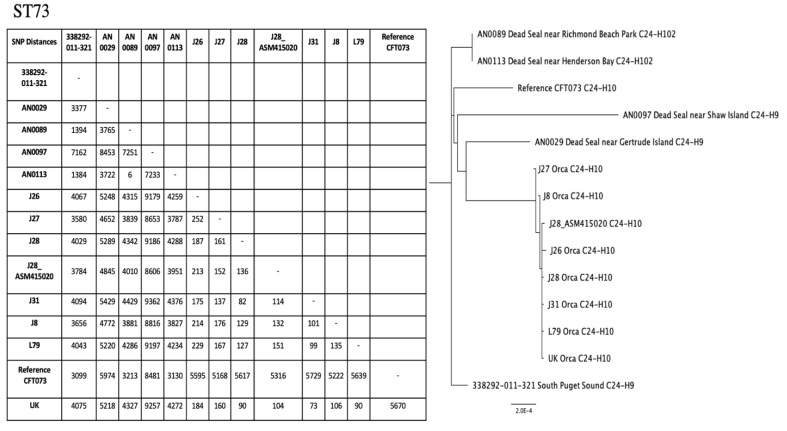
ST10 and ST73 SNP matrices and phylogenetic trees.

Phylogenetic trees were created for ST10 and ST73 (Figure 2). Among ST10 isolates, *fumC:fimH* types included C11:H23, C11:H27, C11:H43, and C11:H54. The single nucleotide polymorphism (SNP) matrix for ST10 showed that the two most closely related isolates, one marine water sample from Central Puget Sound and another from South Puget Sound, differed by 2933 SNPs (Figure 2). The ST73 isolates included two clusters: one from the current study and the other from the previous study with E. coli from Southern Resident killer whales [14]. Two seal fecal samples of ST73, one from Richmond Beach Park in Central Puget Sound and the other from Henderson Bay in South Puget Sound, had a SNP difference of 6 (Figure 2). Both samples shared C24:H102.

### 2.5. Virulence Factors in Nonsusceptible E. coli

Our analysis determined that of the 51 nonsusceptible isolates, three had no virulence factors identified (dead seal sources; AN0041, AN0044, and AN0071), two of which were ST372 isolates. The virulence factor composition was similar, if not identical, among isolates with the same ST (Table 2). The *gad* (glutamate decarboxylase) gene [15] was the most commonly identified virulence factor, appearing in 68% of isolates (*n* = 35).

## 3. Discussion

We found that marine animals were more likely to carry resistant *E. coli* than marine water. Our few fresh water samples also had a high proportion of resistant *E. coli*, but the numbers were too low for statistical analysis. The correlation between phenotypic resistance and genotypic carriage of genes conferring resistance varied with the antibiotic. Tetracycline-resistant/intermediate isolates showed a 100% correlation between phenotype and carriage of a *tet* gene, while aminoglycoside genes did not correlate with phenotypic resistance (Table 2). We also found a disconnect between the results of phenotypic susceptibility testing and the presence or absence of ARGs by WGS analysis for other antibiotics. This could be in part due to incomplete coverage of the WGS so that we did not find complete gene sequences. This is concerning, as more bacteria undergo only WGS and the antibiotic-resistant genes determined by sequencing while the phenotypes are not determined. Thus, we do not know if the gene sequences are functional. What does it mean to clinical medicine if an organism is not phenotypically resistant but carries the gene as identified by WGS? This is a question that has been hard to answer [16,17].

There are several reasons why marine mammals could be good sentinels of environmental antibiotic-resistant genes. River otters, harbor seals, and harbor porpoises share many of the same food sources. Understanding if there is any relationship with the marine mammal food web and the proportion of resistant *E. coli* may shed light onto the origins of resistant *E. coli* in these populations. Future research can assess different species in the food web to better understand the exposure and carriage of ARB in marine mammals and river otters. ARGs that come from livestock or human waste may contaminate the environment and lead to horizontal transfer of genes, risking transmission to human-adapted pathogens. The exposure to pollutants from wastewater treatment plants and agriculture and aquaculture run-off may have potential effects on the ecosystem level; thus, the sampling of animals that inhabit the marine environment may indicate potential health effects on humans [18].

Our finding that fresh water resistant *E. coli* were more common than marine water resistant *E. coli* was not surprising Previous studies have shown that resistant *E. coli* are common in fresh water [9,10], while survival in marine water is dependent on many factors including light and salinity [19]. Meanwhile, high levels of ARB in the marine animals we tested may relate to the more stable environment in the intestinal tract of mammals.

Spatial patterns of the occurrence of resistant *E. coli* in seals could not be assessed due to small sample size. There were more resistant and intermediate *E. coli* found from animal samples, which were primarily taken in the Central and South Salish Sea. There were no nonsusceptible samples found in the Strait of Juan de Fuca. This was not expected due to the proximity to the WWTP in Victoria, BC [20,21]. The susceptibility of bacteria recovered in the Strait of Juan de Fuca may not be fully representative of the bacterial ecology; as there were no samples from seal or porpoises isolated in the Strait of Juan de Fuca. Among the resistant *E. coli* from river otters there was no clear pattern for resistance, and there were no obvious differences in resistance between the superfund site, the suburban area, and the rural area (Figure 1D).

We were unable to address spatial patterns for marine mammals because our opportunistic approach restricted samples in the Salish Sea quadrants. We also selected the *E. coli* for as much variability as possible, which is why we found a large number of ST types. Another limitation of the study was that 254 (83.3%) of the isolates were susceptible, and their potential AMR genes were not examined because of the limited number of AMR genes and mutations found with the intermediate resistant isolates.

## 4. Materials and Methods

### 4.1. Study Setting

The Salish Sea is a large body of marine water shared between Washington State, USA and British Columbia, Canada. Over the past few decades, there has been considerable population growth and residential and business development around the Salish Sea, especially in Washington State [22]. The Salish Sea has a complex estuarine system of interconnected marine waterways and basins, with one major connection (Strait of Juan de Fuca) to the Pacific Ocean. The Salish Sea is used for swimming, fishing, boating, and commercial aquaculture of fish and shellfish. It includes several Superfund sites and receives treated wastewater from WWTP along the shoreline within the USA and Canada [20,21]. Reports have suggested that the Salish Sea contains hot spots for high levels of antibiotic-resistance genes and antibiotic residues that have been identified in local salmon [23,24]. Previously, we have cultured antibiotic-resistant extraintestinal pathogenic (ExPEC) *E. coli* from the feces of the endangered Southern Resident killer whales (*Orcinus orca*) who live in the Salish Sea [14].

### 4.2. E. coli Collection and Isolation

The aim of this study was to characterize antibiotic resistance from a diverse set of *E. coli* isolates collected from marine water, fresh water and marine water along beaches, river otters (*Lontra canadensis*), marine mammals (Harbor seal [*Phoca vitulina*] and harbor porpoise [*Phocoena phocoena*]), and English sole (*Parophrys vetulus*). A total of 551 isolates was collected and characterized by *fumC* to select for variety of different *E. coli* [25].

#### 4.2.1. Freshwater, Marine Water by Beaches, and Marine Water Samples

Freshwater samples were opportunistically collected from Piper’s Creek (Carkeek Park, Seattle, WA, USA) and a beaver pond (Golden Gardens Park, Seattle, WA, USA) during 2019. In total, 100 mL of freshwater was processed using Colilert Standard Quanti-Tray 2000^®^ (IDEXX Laboratories, Westbrook, ME, USA) according to manufacturers’ instructions. A second tray with 1 mg/L cefotaxime (Thermo Fisher Scientific, Pittsburgh, PA, USA) added was used to select for resistant *E. coli*. One isolate with each *fumC* type was included in the study (*n* = 5) (Table 1).

Marine water was sampled at beach sites at 15 cm below the surface at the same time and adjacent to fresh water sampling sites. A 1:10 dilution of marine water (10 mL marine water and 90 mL deionized sterile water) was made using the Colilert Standard Quanti-Tray 2000^®^. Another 1:10 dilution of marine water was made with an addition of 1 mg/L cefotaxime (Thermo Fisher Scientific) using the Colilert Standard Quanti-Tray 2000^®^ (IDEXX Laboratories, Westbrook, ME, USA) (Table 1).

Additional marine water samples provided by the Washington Department of Health (WA DOH) were collected from GPS-located sites associated with shellfish beds as part of the WA DOH Shellfish Growing Program Public Health’s shellfish bed monitoring system for fecal coliform analysis, which follows the Environmental Protection Agency (EPA)’s modified A-1 method [26]. Isolates from four quadrants of the Salish Sea, North Puget Sound, Central Puget Sound, South Puget Sound, and Strait of Juan de Fuca were included with the goal of sampling ~50 *E. coli* isolates from each quadrant (Figure 1A). A total of 212 isolates from the quadrants was selected using *fumC* typing for further characterization (Table 1).

#### 4.2.2. English Sole Samples

English sole were caught during summer 2019 by the WA Marine Resources Division (Washington Department of Fish and Wildlife (WDFW)) as part of annual studies. On the boat, crew removed the stomach and intestinal tract and emptied the contents into a 15 mL sterile conical tube, containing 3 mL of sterile saline. The tubes were placed on ice and transported to the University of Washington laboratory within six h. The samples were vortexed, and 1 mL was placed into 99 mL of sterile water and mixed and then processed using the Colilert Standard Quanti-Tray 2000^®^ (IDEXX Laboratories).

#### 4.2.3. River Otter Samples

River otter feces samples were collected along the Green-Duwamish River in Washington at six otter latrine locations (May 2018 to September 2018) (Figure 1D). Three areas were categorized by their proportions of impervious surface and labeled as: industrial, suburban, and rural. Industrial zone samples were collected at Hamm Creek (7.2 km from the Salish Sea); suburban zone samples at Black River (17.7 km upriver), Green River Natural Resources Area (29.8 km), and Cottonwood Grove (32.2 km); and rural zone samples at Green River Natural Area (61.2 km) (unpublished data, M. Wainstein) (Table 1). One other site, Kenco (8.1 km upstream, industrial zone), did not yield any *E. coli* isolates.

Feces were placed into 50 mL conical tubes, iced, and transported to the University of Washington laboratory. Two mL centrifuge tubes were filled with feces to the 0.5 mL mark, and then 0.85% sterile saline was added to the 1.5 mL mark, and the sample was vortexed. A total of 0.1 mL of mixture was plated onto eosin methylene blue (EMB) agar plates (Becton Dickinson, Franklin Lakes, NJ, USA). Other EMB plates were supplemented with one the following antibiotics per plate: ampicillin 25 mg/mL, kanamycin 25 mg/mL, sulfisoxazole 256 mg/mL, spectinomycin 100 mg/mL, streptomycin 100 mg/mL, and tetracycline 25 mg/mL (Fisher Bioreagents, Pittsburgh, PA, USA). Plates were incubated at 36.5 °C overnight.

#### 4.2.4. Marine Mammal Samples

Fecal swabs were collected post mortem from harbor seals and harbor porpoises and processed by Phoenix Lab (Zoetis Reference Labs, Mukilteo, WA, USA) between fall 2018 and fall 2019. Detailed methods and isolates were described previously [27].

All but one live harbor seal fecal samples were collected by WDFW staff from docks at various locations throughout the Puget Sound (Figure 1A), where harbor seals haul out and defecate. The fecal samples were collected with a wooden tongue depressor, transferred to a Whirl-Pak bag, and submitted to University of Washington laboratory within six hours. A single fecal sample was obtained from a stranded seal taken to Progressive Animal Welfare Society Wildlife Center (Lynnwood, WA, USA). A pellet of the feces from all live marine mammals was added to a Durham tube with Brilliant Green Broth (Fisher Bioreagents, Pittsburgh, PA, USA) and incubated at 37 °C overnight. Positive Brilliant Green Broth samples had their *E. coli* verified on an EMB agar plate (Fisher Bioreagents, Pittsburgh, PA, USA). Seventeen *E. coli* were included from live seal fecal samples (Table 1).

### 4.3. fumC Typing

A previous study found that *fumC*, one of the genes used for MLST typing, could be used to type extraintestinal pathogenic *E. coli* [25]. Therefore, it was used to help us determine different strains of *E. coli* from the same samples because we wanted to examine the most diverse *E. coli* population for this study. This worked very well as illustrated from different MLST identified in Table 2. Thus, different *fumC* types were selected from isolates collected in the same general location and time period in order to prevent duplicate isolates (see below). The *fumC* PCR assay was performed with published primers as previously described, using *E. coli* MG1655 as a positive control [25]. PCR products were sequenced at Eurofins Genomics (Louisville, KY, USA). The sequences were edited, aligned, and compared with the Achtman MLST database (https://pubmlst.org/bigsdb?db=pubmlst_mlst_seqdef&page=schemeInfo&scheme_id=4 accessed on 30 March 2021) to determine the *fumC* from PCR products or directly from WGS (see below).

### 4.4. Antimicrobial-Susceptibility Testing

#### 4.4.1. Phenotypic Characterization

The marine water (WA DOH), fish, and live seal *E. coli* isolates were analyzed using broth dilution antibiotic susceptibility testing with the Sensititre^TM^ Nephelometer (Thermo Fisher Scientific, Waltham, MA, USA) according to manufacturer’s Sensititre AIM instructions at the WA DOH laboratory. The panels were read using Sensititre SWIN software and were also inspected visually for microbial growth. The minimum inhibitory concentration (MIC) for each antibiotic in mg/mL using the CLSI interpretive criteria (Clinical and Laboratory Standards Institute, 2021) [28] determined if isolates were susceptible, intermediate resistant, or resistant to the following antibiotics: amikacin, aztreonam, cefepime, cefotaxime, ceftazidime, ciprofloxacin, doripenem, doxycycline, ertapenem, gentamicin, imipenem, levofloxacin, meropenem, minocycline, piperacillin/tazobactam, ticarcillin/clavulanic acid, tigecycline, tobramycin, and trimethoprim/sulfamethoxazole. Standard positive and negative controls for *E. coli* were used.

*E. coli* from dead seals and porpoises were tested using the bioMérieux VIETK instrument (Durham, NC, USA). The *E. coli* isolated from river otters, fresh water, marine water by beaches, and the rescued seal pup were tested using a standard disk diffusion assay according to CLSI [28]. Standard *E. coli* negative and positive controls were included each assay.

#### 4.4.2. Genotypic Characterization

WGS was performed on the 305 *E. coli* isolates as part of the *E. coli* GenomeTrakr Project of WA DOH (ID 283914-BioProject-NCBI), using Illumina (Illumina, San Diego, CA, USA) [29]. MLST were determined from the sequence data [7]. Sequences are maintained by the National Center for Biotechnology Information (NCBI) and assigned an accession number and SRR ID. NCBI Accession Numbers [SAMN]: 13337618, 13348248, 13352752, 13352855–13352864, 13392846, 13392848–13392863, 13392951–13392953, 13418005, 13429240, 13429289, 13482430, 13502693, 13502695, 13502889–13502891, 13513927–13513929, 13513928–13513930, 13513935–13513938, 13513942, 13513948, 13518346, 13518347, 13898866–13898880, 13911824, 13911825, 14057293, 14057294, 14080880–14080885, 14083856–14083863, 14083865, 14083866, 14083868–14083870, 14083873, 14084247, 14113834, 14113836–14113844, 14113847, 14113850, 14113860–14113863, 14137883–14137888, 14137890–14137892, 14137896–14137905, 14137905, 14137978, 14137979, 14138286, 14140185–14140189, 14140195–14140217, 14214490–14214498, 14270850–14270852, 14271025, 14271030–14271033, 14291765, 14316584–14316586, 14316588–14316590, 14316618, 14316619, 14316621, 14316622, 14316624, 14316625, 14316627, 14316629, 14316633, 14316684–14316687, 14593716–14593722, 14749987, 14749988, 14749995, 14750012, 14750852, 14750854–14750856, 15182299–15182304, 15182308, 15182310–15182316, 15182319, 15182320, 15182323, 15344667, 15344671, 15344672, 15344674, 15483654–15483656, 15777149, 15777151, 15777153–15777155, 15777158, 15777160, 15777162, 15777164, 15777165, 15777167, 16054328–16054337, 16054339–16054347, 16054538, 16056701–16056705, 16056743–16056748, 16136466, 16136468, 16136469, 16136474–16136479, 16136481, 16136482, 16136485, 16136487, 16136489, 16136490, 16202553–16202558, 16257942–16257946, 16377217–16377219, and 16439289.

### 4.5. Comparison of Susceptibility Rates

We evaluated antibiotic-susceptibility proportions within three *E. coli* isolate groupings, comparing (1) isolates among the four quadrants of the Salish Sea; (2) marine water versus all wildlife isolates (river otter, harbor porpoise, and harbor seal); and (3) marine water versus only marine mammal isolates (excluding river otters). Sample sizes for fresh water and marine water by beaches were too small to be included in the analyses. For each isolate grouping, we made two comparisons: susceptible versus resistant and susceptible versus nonsusceptible (resistant and intermediate). For each comparison, the Fisher’s exact test was used and Bonferroni-adjusted for repeated measures. A confidence level of 0.05 was selected and Bonferroni-adjusted for the six different hypothesis tests (α = 0.05/6 = 0.008 = *p*-value). Statistical analysis was conducted using R version 3.6.1.

### 4.6. Phylogenetic Trees

Phylogenetic trees were created in the University of Washington Department of Environmental and Occupational Health Sciences Linux Environment using the SRR ID generated by WGS. The raw sequencing files for the isolates were downloaded as FASTQ files into Plasmid. The program Trimmomatic [30] cleaned the FASTQ files by removing the Ilumina adapters and prepared the files for alignment against a reference genome and strains of the same ST from our data using the program, Snippy [31]. Human reference genomes were selected based on the ST and *fumC:fimH* (CH) type from NCBI GenBank. Human reference for ST10 was RS218, a ST95 newborn meningitis strain, and MG1655, laboratory K12 strain, and the human reference for ST73 was CFT073. Once the isolates were aligned with the reference strain, the program SNP-DISTS [32] created the single nucleotide polymorphism (SNP) difference matrix to analyze SNP differences between isolates with the same ST. An alignment file created by SNP-DISTS was converted into a ‘.phy’ file by AliView [33]. The ‘.phy’ file was converted into the appropriate format by Phylip [34] in order to use the in the program FigTree to create phylogenetic trees [35].

### 4.7. Mapping

Mapping was performed using QGIS, version 3.2.3. Several maps were created by sample location (Figure 1A) to visually identify if any clusters existed based on nonsusceptibility (Figure 1B), ExPEC ST (Figure 1C), and river otter sample source (Figure 1D).

### 4.8. Antimicrobial Resistance (AMR) Genes and Virulence Factor Analysis

FASTQ files for phenotypically resistant and intermediate isolates were analyzed using ResFinder [36]. We selected *E. coli*, choosing to show only known mutations and all acquired antimicrobial configurations, using a 90% threshold and 100% minimum length for both selections.

VirulenceFinder [37] was used to identify the virulence factors of intermediate and resistant isolates. We selected *E. coli*, using a 90% threshold and 100% minimum length, of the raw sequencing reads.

## 5. Conclusions

There was no statistical difference in the proportion of resistance and nonsusceptibility *E. coli* when comparing the four quadrants of the Puget Sound. This could be due to relatively low numbers taken at each quadrant. When comparing the proportion of resistance and nonsusceptibility to susceptibility in our mammal samples and marine water samples, our analysis determined that there was a higher proportion of resistant and intermediate isolates taken from animal sources with significant *p*-value (*p* < 0.0001). Looking solely at the difference of proportion of resistant and intermediate isolates in marine mammals and marine water, the analysis determined that there was a higher number of nonsusceptible isolates, when the *E. coli* came from a marine mammal source (*p* = 0.005). There was not an overwhelming spatial clustering of antibiotic-resistant *E. coli* potentially due to the total distribution of marine mammals. We would have expected to see more in the strait due to the secondary WWTP near Vancouver Island, but there were few marine mammal samples found in that region. We had the assumption that we would find a lot of resistant *E. coli* within more urban or agricultural areas, but we were limited due to our sampling methods. We observed clustering of resistant *E. coli* that correlated with where marine mammals and river otters were sampled. Our isolates from river otters were samples along a 56 km river complex starting with the Lower Duwamish superfund site and ending with a rural area. We found resistant and intermediate isolates along the length of where we sampled. Using WGS to characterize our isolates, we found that there was a diverse number of STs found in our samples and that ExPEC ST were present in the animal and water samples. There were very few clones which came from similar locations and sources, and none of the isolates were closely related to human isolates. More work needs to be conducted to determine if antibiotic-resistant *E. coli* are also found in mammals in other water ecosystems and if there are changes in levels of resistance over time. Future research will look at human isolates in the region to better understand the flow of resistant *E. coli* in this ecosystem.

## Figures and Tables

**Table 1:** Total number of isolates characterized and antibiotic-susceptibility testing results for each source.

Sample Source	Isolates Characterized	Intermediate	Resistant	Susceptible
Marine Water (Total)	212	7 (3.3%)	7 (3.3%)	198 (93.4%)
North Puget Sound	49	3 (6.1%)	4 (8.2%)	42 (85.7%)
Central Puget Sound	55	0 (0%)	2 (3.6%)	53 (96.4%)
South Puget Sound	56	3 (5.4%)	0 (0%)	53 (94.6%)
Strait of Juan de Fuca	52	1 (1.9%)	1 (1.9%)	50 (96.2%)
Freshwater	5	1 (20%)	3 (60.0%)	1 (20.0%)
Marine water by beaches	3	0 (0%)	0 (0%)	3 (100%)
Harbor Seal (Total)	52	6 (11.5%)	8 (15.4%)	38 (73.1%)
Dead Seal	35	6 (17.1%)	3 (8.6%)	26 (74.3%)
Live Seal	17	0 (0%)	5 (29.4%)	12 (70.6%)
Harbor Porpoise	7	2 (28.6%)	0 (0%)	5 (71.4%)
River Otter	24	4 (16.7%)	13 (54.2%)	7 (29.2%)
Sole	2	0 (0%)	0 (0%)	2 (100%)
Total	305	20 (6.6%)	31 (10.2%)	254 (83.3%)

**Table 2:** The 51 nonsusceptible *E. coli* antibiotic phenotype, genotype, and virulence genes.

Isolate ID	Source	MLST	Resistance Phenotype	Resistance Phenotype by Antibiotic	Resistant Genes by WGS ^a^	Virulence Factors ^a^
353985-001-1210	South Puget Sound	2	Intermediate	Imipenem (Intermediate)	None	*ast, chuA, lpfA*
339942-001-501	North Puget Sound	10	Resistant	Minocycline, SXT ^b^	*qnrB19, sulIII, dfrA12, floR, tet*(A*)*	*gad, terC*
HAM6D	River Otter	10	Resistant	Ampicillin, SXT, Tetracycline	*aph(6)-Id, bla*_TEM-1B_*, tet*(B)	*astA, cia, gad, terC, traT*
CWG3I	River Otter	10	Resistant	Cefotaxime (Intermediate), Tetracycline, Minocycline (Intermediate), Sulfisoxazole (Intermediate)	*tet*(B)	*gad, kpsE, kpsM* II*, terC*
344914-013-1036	Central Puget Sound	58	Resistant	Doxycycline, Minocycline (Intermediate)	*tet*(B), *aph(3″)-Ib, aph(6)-Id*	*cia, cvaC, etsC, fyuA, gad, hlyF, iroN, iss, iucC, iutA, lpfA, chF, ompT, terC, traT*
339942-002-506	North Puget Sound	58	Resistant	Aztreonam, Cefotaxime, Doxycycline, SXT, Ciprofloxacin (Intermediate)	*sulIII, dfrA12, tet*(A), *floR, bla*_CTX-M-15_*, qnrS1, qnrB19*	*gad, hlyF, lpfA, terC*
HAM5E	River Otter	69	Resistant	Ampicillin, SXT, Tetracycline, Minocycline, Sulfisoxazole	*aadA5, aph(3″)-Ib, aph(6)-Id, bla*_TEM-1B_*, catA1, qnrB19, qnrB82, sulII, tet*(B), *dfrA17*	*air, chuA, eilA, fyuA, gad, hra, iha, irp2, iucC, lutA, kpsE,* *kpsM* II_K52*, lpfA, ompT, papA, fsiA*(F16)*, papC, sat, senB, traT*
SSW080719 (AN0077)	Dead Seal	117	Resistant	Doxycycline	*tet*(B), *sulII, aph(6)-Id, aph(3″)-Ib, aph(3′)-Ia*	*astA, chuA, etsC, fyuA, hlyF, hra, iroN, irp2, iss, lucC, ompT, pic, traT, vat*
SSW082919 (AN0092)	Dead Seal	117	Resistant	Doxycycline	*tet*(B), *sulII, aph(6)-Id, aph(3″)-Ib, aph(3′)-Ia*	*astA, chuA, etsC, fyuA, hlyF, hra, iroN, irp2, iss, lucC, ompT, pic, traT, vat*
WDFW2019-154 (AN0107)	Dead Seal	131	Resistant	Amoxicillin, Gentamicin, SXT	*aac(3)-Iid, aadA2, dfrA12, sulI, mph*(A)*, bla*_TEM-1B_	*afaA, afaC, afaD, afaE, chuA, fyuA, gad, iha, irp2, iss, iucC, iutA, kpsE,* *kpsM* II_K5*, ompT, sat, senB, traT, yfcV*
343170-001-909	North Puget Sound	131	Intermediate	Ciprofloxacin (Intermediate), Ticarcillin/Clavulanic Acid (Intermediate)	*bla*_TEM-1B_*, gyrA* (S83L)	*afaA, afaD, chuA, fyuA, gad, kpsE, kpsM* II_K5*, ompT, senB, traT, yfcV*
GRNRA2B	River Otter	131	Resistant	Ampicillin, Imipenem (Intermediate), Kanamycin (Intermediate), Sulfisoxazole (Intermediate)	*bla*_TEM-1C_	*chuA, gad, ibeA, irp2, iss,* *kpsM* II, *papA_F48, sitA, yfcV*
WDFW2019-107 (AN0070)	Dead Seal	162	Intermediate	Florfenicol (Intermediate), Chloramphenicol (Intermediate)	None	*gad, lpfA, terC, traT*
CWG7G	River Otter	162	Resistant	Sulfisoxazole, Cefotaxime (Intermediate), Amikacin (Intermediate), Kanamycin (Intermediate)	None	*gad, hlyF, iss, iucC, iutA, lpfA, terC*
CWG7H	River Otter	162	Resistant	Ampicillin (Intermediate), Amikacin (Intermediate), Kanamycin (Intermediate), Sulfisoxazole	None	*gad, hlyF, lucC, lutA, lpfA,* *terC*
342381-006-850	Strait of Juan de Fuca	206	Resistant	Aztreonam, Cefotaxime, Ceftazidime	None	*astA, gad, traT*
PCB4Cef	Fresh Water	297	Resistant	Ampicillin, Amoxicillin/Clavulanic Acid, Ceftriaxone, Aztreonam, Ceftazidime, Ticarcillin/Clavulanic Acid (Intermediate)	*bla*_CMY-2_	*cib, gad, lpfA, mchB*
SKMMR2020-01-025 Gut #1	Live Seal	345	Resistant	SXT	*dfrA5*	*cia, cvaC, etsC, gad, hlyF, iroN, iss, lpfA, ompT, sitA*
GRNRA3B	River Otter	362	Intermediate	Cefotaxime (Intermediate), Sulfisoxazole (Intermediate)	None	*chuA, iss, kpsE,* *kpsM* II_K5
GRNRA4A	River Otter	362	Intermediate	Cefotaxime (Intermediate), Imipenem (Intermediate), Meropenem (Intermediate), Amikacin (Intermediate), Kanamycin (Intermediate), Sulfisoxazole (Intermediate)	*qnrB19*	*chuA, iss, kpsE,* *kpsM* II_K5
GRNRA4B	River Otter	362	Resistant	Sulfisoxazole, Cefotaxime (Intermediate), Imipenem (Intermediate), Meropenem (Intermediate), Kanamycin (Intermediate), Ciprofloxacin (Intermediate)	None	*chuA, iss, kpsE,* *kpsM* I_K5
SKMMR2019-7-10PV (AN0044)	Dead Seal	372	Intermediate	Florfenicol (Intermediate)	None	None
19Pv16JulWI-07 Isolate #1 (AN0047)	Dead Seal	372	Intermediate	Florfenicol (Intermediate)	None	*cea, focC, sfaE, focG, focI, fyuA, gad, hra, ibeA, iroN, irp2, iss kpsE,* *kpsM* II_K24, *mchB, mchF, ompT, papA_F13, terC*
19Pv29JulWI-09 Isolate #2 (AN0041)	Dead Seal	372	Intermediate	Florfenicol (Intermediate), Amoxicillin (Intermediate)	None	None
GG 14-6 Cef	Fresh Water	405	Resistant	Aztreonam, Cefepime, Cefotaxime, Ceftazidime, Ciprofloxacin, Doxycycline, Levofloxacin, Minocycline, Ticarcillin/Clavulanic Acid, SXT	*sulI, mph*(A)*, bla*_CTX-M-15_*, aadA2, qepA4, dfrA12, catA1, tet*(B), *qepA, gyrA* S83L*, gyrA* D87N	*chuA, fyuA, irp2,* *kpsM* II_K5*, sitA, traT*
GRNRA2E	River Otter	538	Resistant	Cefotaxime, Sulfisoxazole, Ampicillin (Intermediate), Imipenem (Intermediate), Meropenem (Intermediate), Amikacin (Intermediate)	*aac(2′)-Iia*	*ibeA, neuC, ompT*
CRC-1702 (AN0006)	Porpoise	569	Intermediate	Florfenicol (Intermediate) Chloramphenicol (Intermediate)	None	*chuA, fyuA, ibeA, iss kpsE* *kpsM* II_K1*, neuC, ompT, sitA, usp*
GG 14-5 Cef	Fresh Water	616	Resistant	Aztreonam, Cefotaxime, Ceftazidime (Intermediate), Cefepime	*bla*_CTX-M-15_*, qnrS1, mph*(A)	*gad, terC, traT*
343066-013-868	South Puget Sound	641	Intermediate	Aztreonam (Intermediate)	None	*gad, lpfA, ompT, traT*
PCO1	Fresh Water	681	Intermediate	Ceftriaxone (Intermediate)	None	*chuA, cia, cibB, iss, ompT, traT*
EPA Dock G Cip 1#5	Live Seal	744	Resistant	Ciprofloxacin, Doxycycline (Intermediate), Levofloxacin	*aph(3″)-Ib, aph(6)-Id, catA1, floR, sulII, tet*(A), *gyrA* S83L, *gyrA* D87N	*gad*
SKMMR2020-01-025 Fecal #1	Live Seal	744	Resistant	Ciprofloxacin, Levofloxacin	*aph(3″)-Ib, aph(6)-Id, mdf*(A), *catA1, floR, sulII, tet*(A)*, gyrA* S83L*, gyrA* D87N	*gad*
351565-001-1202	North Puget Sound	744	Resistant	Ciprofloxacin, Doxycycline, Levofloxacin, Minocycline, SXT	*sulI, dfrA17, tet*(A), *sulII, tet*(B), *bla*_TEM-1B_*, aph(3″)-Ib, mph*(A), *aadA5, catA1, aph(6)-Id, gyrA* S83L*, gyrA* D87N	*cvaC, etsC, gad, hlyF, iroN, iss, mchF, traT*
339942-003-511	North Puget Sound	746	Resistant	Cefotaxime, Doxycycline (Intermediate), Gentamicin (Intermediate)	*aac(3)-Via, aph(3″)-Ib, aadA1, aph(6)-Id, sulI, bla*_SHV-12,_*tet*(A)	*cib, cma, fyuA, gad, hlyF, iroN, irp2, iss, neuC, terC, traT*
EPA Dock G#1	Live Seal	772	Resistant	Doxycycline, SXT, Minocycline (Intermediate)	*aadA5, sulII, tet*(B), *dfrA17*	*cma, gad, irp2, terC*
343389-008-981	North Puget Sound	942	Intermediate	Amikacin (Intermediate), Ticarcillin/Clavulanic Acid (Intermediate)	None	*lpfA, sitA, terC*
354777-001-1214	Strait of Juan de Fuca	967	Intermediate	Aztreonam (Intermediate)	None	*cba, chuA, cma, ibeA,* *kpsM* II_K5
BR1F	River Otter	1079	Resistant	Ampicillin, Gentamicin, Tetracycline, Minocycline	*aac(3)-IV, aac(3)-Iva, aadA1, aph(4)-Ia, aph(6)-Id, bla*_TEM-1B_*, lnu*(F), *tet*(B)	*gad, lpfA, terC*
BR1E	River Otter	1079	Resistant	Doxycycline, Gentamicin, Tobramycin, Minocycline (Intermediate)	*aac(3)-IV, aph(4)-Ia, aph(6)-Id, bla*_TEM-1B_*, lnu*(F), *tet*(B)	*gad, lpfA, terC*
GRN1A	River Otter	1246	Intermediate	Ampicillin (Intermediate), Sulfisoxazole (Intermediate)	None	*gad, lpfA, terC*
2019-SJ013 (AN0032)	Dead Seal	1718	Intermediate	Florfenicol (Intermediate)	None	*gad, terC*
EJC-2019-03 (AN0009)	Porpoise	1723	Intermediate	Florfenicol (Intermediate), Amoxicillin (Intermediate), Chloramphenicol (Intermediate)	None	*cma, gad, ipfA, traT*
CWG3J	River Otter	2144	Resistant	Chloramphenicol, Tetracycline, Sulfisoxazole, Minocycline (Intermediate)	*aadA1, cmlA1, sulIII, tet*(A)	*cib, gad, lpfA, ompT*
GRNRA2F	River Otter	2164	Resistant	Cefotaxime, Imipenem, Meropenem (Intermediate), Kanamycin (Intermediate), Sulfisoxazole (Intermediate)	None	*gad, iss, lpfA, ompT, terC*
GRNRA4F	River Otter	2521	Resistant	Sulfisoxazole, Cefotaxime (Intermediate), Ampicillin (Intermediate), Imipenem (Intermediate), Meropenem (Intermediate), Kanamycin (Intermediate)	None	*gad, iss, lpfA, ompT, terC*
345996-003-1186	North Puget Sound	2522	Intermediate	Aztreonam (Intermediate)	None	*gad, lpfA*
CWG5A	River Otter	2607	Intermediate	Cefotaxime (Intermediate), Imipenem (Intermediate), Kanamycin (Intermediate)	None	*gad, lss, lpfA, ompT, terC*
WDFW2019-112 (AN0071)	Dead Seal	3018	Intermediate	Florfenicol (Intermediate)	None	None
336039-006-31	South Puget Sound	7706	Intermediate	Ciprofloxacin (Intermediate)	None	*gad, iss*
HASE 6 CEF	Live Seal	9001	Resistant	Ampicillin, Amoxicillin/Clavulanic Acid, Ceftriaxone, Aztreonam, Cefotaxime, Ceftazidime, Ticarcillin/Clavulanic Acid (Intermediate)	*bla*_CMY-2_	*astA, hlyF, hra, traT*
339940-002-477	Central Puget Sound	10718	Resistant	Cefotaxime, Ceftazidime, Ticarcillin/Clavulanic Acid (Intermediate)	*bla*_CMY-2_	*gad, lpfA, ompT, terC*

^a^ As found by ResFinder 4.0 and VirulenceFinder, ^b^ SXT abbreviation for Trimethoprim/Sulfamethoxazole.

**Table 3:** Locations and counts of resistant isolates for each ExPECS.

Sample Source (*n* = 37)	ST10	ST10 Resistant	ST38	ST38 Resistant	ST58	ST58 Resistant	ST69	ST69 Resistant	ST73	ST73 Resistant	ST117	ST117 Resistant	ST131	ST131 Resistant	ST405	ST405 Resistant	Total
Marine Water (Total)	10	1	2	0	2	2	2	0	1	0	3	0	1	0	0	0	21
North Puget Sound	4	1	0	0	1	1	1	0	0	0	0	0	1	0	0	0	7
Central Puget Sound	4	0	0	0	1	1	0	0	0	0	1	0	0	0	0	0	6
South Puget Sound	1	0	2	0	0	0	0	0	1	0	0	0	0	0	0	0	4
Strait of Juan de Fuca	1	0	0	0	0	0	1	0	0	0	2	0	0	0	0	0	4
Fresh water	0	0	0	0	0	0	0	0	0	0	0	0	0	0	1	1	1
Marine water by beaches	0	0	0	0	1	0	0	0	0	0	1	0	0	0	0	0	2
Harbor Seal (Total)	0	0	0	0	1	0	0	0	4	0	2	2	1	0	0	0	8
Dead Seal	0	0	0	0	1	0	0	0	4	0	2	2	1	1	0	0	8
Live Seal	0	0	0	0	0	0	0	0	0	0	0	0	0	0	0	0	0
Harbor Porpoise	0	0	0	0	0	0	0	0	0	0	0	0	0	0	0	0	0
River Otter	2	2	0	0	0	0	2	1	0	0	0	0	1	1	0	0	5
Sole	0	0	0	0	0	0	0	0	0	0	0	0	0	0	0	0	0
**Total**	**12**	**3**	**2**	**0**	**4**	**2**	**4**	**1**	**5**	**0**	**6**	**2**	**3**	**2**	**1**	**1**	**37**

## Data Availability

All genomic data related to this project are available via NCBI GenBank under project 283914-BioProject.
